# Genome Sequence of *Fusarium oxysporum* f. sp. *melonis* Strain NRRL 26406, a Fungus Causing Wilt Disease on Melon

**DOI:** 10.1128/genomeA.00730-14

**Published:** 2014-07-31

**Authors:** Li-Jun Ma, Terrance Shea, Sarah Young, Qiandong Zeng, H. Corby Kistler

**Affiliations:** a Department of Biochemistry and Molecular Biology, University of Massachusetts Amherst, Amherst, Massachusetts, USA; b Broad Institute of Harvard and MIT, Cambridge, Massachusetts, USA; c United States Department of Agriculture, ARS Cereal Disease Laboratory, University of Minnesota, St. Paul, Minnesota, USA

## Abstract

Horizontal chromosome transfer introduces host-specific pathogenicity among members of the *Fusarium oxysporum* species complex and is responsible for some of the most destructive and intractable plant diseases. This paper reports the genome sequence of *F. oxysporum* f. sp. *melonis* (NRRL 26406), a causal agent of *Fusarium* wilt disease on melon.

## GENOME ANNOUNCEMENT

Collectively, the genus *Fusarium* represents the most important group of fungal plant pathogens, causing various diseases on nearly every economically important plant species. Of equal concern is the health hazard posed to humans and livestock by the plethora of *Fusarium* mycotoxins (1). Besides their economic importance, species of *Fusarium* also serve as key model organisms for biological and evolutionary research (2).

Members of the *Fusarium oxysporum* species complex exhibit extraordinary genetic plasticity and cause some of the most destructive and intractable diseases across a diverse spectrum of hosts, including many economically important crops, such as bananas, cotton, canola, melons, and tomatoes. *Fusarium* comparative genomics has revealed that horizontal chromosome transfer introduces host-specific pathogenicity among members of this species complex and is responsible for the broad host range and the strong host specificity revealed by the members within the *F. oxysporum* species complex (3).

This paper reports the genome sequence of *F. oxysporum* f. sp. *melonis*, a fungal pathogen that causes *Fusarium* wilt disease on melon (*Cucumis melo*). The project is part of a large comparative study designed to explore the genetic composition and evolutionary origin of this group of horizontally transferred chromosomes among a set of selected strains that capture the pathogenic and phenotypic diversity of the species complex.

The total genomic DNA was extracted from *F. oxysporum* f. sp. *melonis* strain NRRL 26406, a field isolate originally collected from Mexico. The strain was deposited and available at the USDA Agricultural Research Service (ARS) Culture Collection. More than 150-fold sequence coverage and >100 physical coverage sequences were generated from two libraries using Illumina sequencing technology, resulting in a 180-base fragment and 3-kb jumping libraries. The assembly was generated using AllPaths-LG (version R37753), run with default parameters (4). Mitochondrial sequences were removed by searching against an NCBI mitochondrial database. The genome size was estimated to be 68 Mb based on the *k*-mer frequency of the initial reads using Kmer spectrum, a module run within AllPaths-LG (4). The assembled genome size is 54.03 Mb, with a G+C content of 47.5%. The discrepancy in the estimated genome size and the assembled genome is largely due to the highly repetitive nature of this genome. More than 28% of the read data may be considered repetitive based on the copy number (CN) of the constituent *k*-mers. The assembly is organized in 1,832 contigs in 1,152 scaffolds. The average base is found in a scaffold with an *N*_50_ of 2.2 Mb and a contig with an *N*_50_ of 430 kb.

This genome contains a total of 61 rRNA, 311 tRNA, and 20,033 protein-coding genes. *Ab initio* gene models were created combining predictions from GeneMark-ES, GeneId, Augustus, GlimmerHMM, and SNAP, in conjunction with strand-specific PASA alignment and GeneWise features from BLAST against the UniRef90 database. The gene models were further updated with RNA-Seq datasets. The resulting annotation was filtered to remove spurious genes that overlap with transposons.

### Nucleotide sequence accession number.

The whole-genome sequence and annotation of *F. oxysporum* f. sp. *melonis* strain NRRL 26406 have been deposited at DDBJ/EMBL/GenBank under the accession no. AGNE00000000.1.
